# *Per aspera ad astra*: When harmful chromosomal translocations become a plus value in genetic evolution. Lessons from *Saccharomyces cerevisiae*.

**DOI:** 10.15698/mic2015.10.230

**Published:** 2015-08-20

**Authors:** Valentina Tosato, Carlo V. Bruschi

**Affiliations:** 1 Yeast Molecular Genetics Laboratory, International Centre for Genetic Engineering and Biotechnology, Trieste, Italy.

**Keywords:** adaptation, BIT, chromosomal translocation, evolution, rearrangements, yeast

## Abstract

In this review we will focus on chromosomal translocations (either spontaneous or induced) in budding yeast. Indeed, very few organisms tolerate so well aneuploidy like *Saccharomyces*, allowing in depth studies on chromosomal numerical aberrations. Many wild type strains naturally develop chromosomal rearrangements while adapting to different environmental conditions. Translocations, in particular, are valuable not only because they naturally drive species evolution, but because they might allow the artificial generation of new strains that can be optimized for industrial purposes. In this area, several methodologies to artificially trigger chromosomal translocations have been conceived in the past years, such as the chromosomal fragmentation vector (CFV) technique, the Cre-*loxP* procedure, the FLP/*FRT* recombination method and, recently, the bridge - induced translocation (BIT) system. An overview of the methodologies to generate chromosomal translocations in yeast will be presented and discussed considering advantages and drawbacks of each technology, focusing in particular on the recent BIT system. Translocants are important for clinical studies because translocated yeast cells resemble cancer cells from morphological and physiological points of view and because the translocation event ensues in a transcriptional de-regulation with a subsequent multi-factorial genetic adaptation to new, selective environmental conditions. The phenomenon of post-translocational adaptation (PTA) is discussed, providing some new unpublished data and proposing the hypothesis that translocations may drive evolution through adaptive genetic selection.

## INTRODUCTION

Adaptive evolution emerges through random genomic mutations that usually reduce and rarely improve the fitness of an organism. *Saccharomyces cerevisiae* for its genomic plasticity, aneuploidy tolerance and finest recombination machinery is the ideal model organism to study chromosome changes selectively established during environmental adaptation. The majority of these genomic variations are limited to single nucleotide polymorphisms, intra-chromosomal gene copy number duplication, and difference in sub-telomeric lengths. However, recent studies on industrial strains of *S. cerevisae *used for biofuel and wine production, suggested that massive alterations in gene transcription were associated with adaptive evolution during long-term fermentations. Genomic analysis of these new strains revealed that spontaneous gross chromosomal rearrangements and in particular chromosomal translocations were responsible for this transcriptional shift and were due to adaptation to sulfites 1 and to high ethanol concentration 2,3. Unexpectedly, also the variation of fermentation rates was associated with aneuploidies such as a partial disomy of chromosome XVI, resulting from a VIII/XVI translocation 4. All these observations suggest that yeast has to cope with multiple stresses generated by wine fermentation and adapts developing new rearrangements with consequent acquisition of resistance 4. It is not so predictable and noticeable that adaptation could be associated with translocations in budding yeast because its genome, despite the plasticity, maintains easily the ploidy state and chromosome organization, differently from other fungi, such as the closely related pathogen *Candida glabrata*, which shows high-occurrence of spontaneous chromosome translocation in mitosis 5. Particular types of rearrangements have been characterized such as translocations recovered in aged *S. cerevisiae* strains following 100-500 generations of growth in glucose-limited chemostats 6 and the ones resulting from recombination between transposable Ty elements 7; these studies are necessary to justify genetic differences among yeast strains although not sufficient models to describe spontaneous and rare rearrangement events.

In order to understand the control of genome stability and adaptive evolution and to have valid tools to manipulate the yeast genome, several biological methods have been developed. Ploidy maintenance was altered through many systems such as the FLP-*FRT* 8, the* Cre-loxP* 9, the Gin-Gix systems 10, the R-Rs 11 and the chromosomal fragmentation vector technology 12*.* In most of the cases, the initial double strand break (DSB) is generated thanks to the I-SceI or the HO endonuclease. More recently, the BIT system was developed, which allows the formation of chromosomal translocations without pre-engineering of the genome. All these GCRs (gross chromosomal rearrangement) - providing methodologies are based on homology or micro-homology recombination and will be analyzed in details in the next paragraphs. Non-homologous end joining - based techniques and physical/chemical approaches will not be surveyed in this review.

## ENZYME-DEPENDENT, SITE-SPECIFIC RECOMBINATION TECHNOLOGIES TO INDUCE REARRANGEMENTS IN THE YEAST GENOME

The site-specific recombination systems most commonly used in yeast as well as in higher eukaryotes are the FLP/*FRT* and the Cre/*loxP *techniques.

**Figure 1:**
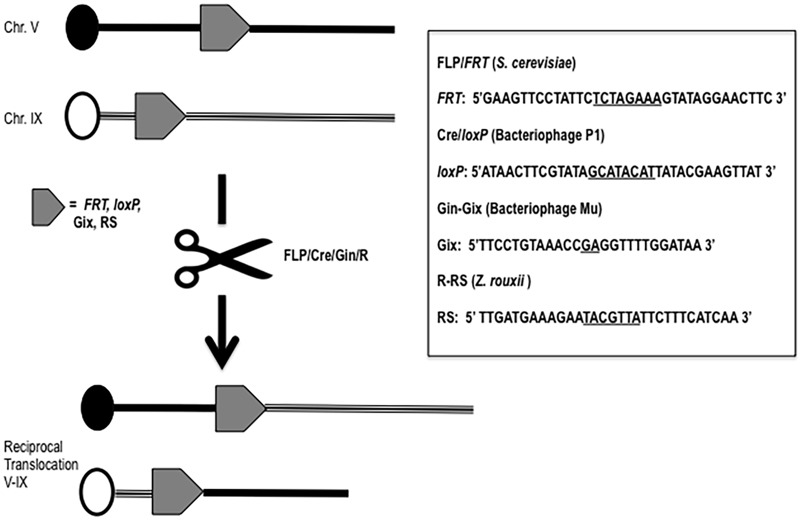
FIGURE 1: Scheme summarizing a reciprocal translocation event promoted by the most used recombinases. On the right, their recognition target sequences are reported. The asymmetric core regions are underlined.

The first is based on 2µ plasmid, a circular DNA molecule of 6,318 bp present in about 40-60 copies per haploid cell in most yeast strains. Recombination between two 599-bp DNA inverted repeats of the plasmid, mediated by its own encoded FLP-recombinase, generates the inversion of a segment of the DNA sequence of the plasmid, leading to two isoforms, A and B, that are found in equal concentrations in yeast cells. No cellular function has been so far elucidated for 2µ and defining its unknown role will be one of the great challenges for the yeast scientists in the next years. However, this selfish plasmid provides one of the most efficient recombination system ever discovered: the FLP/*FRT* 8. The FLP recombinase catalyzes the recombination of two 13-bp repeats separated by a 8-bp spacer in a 34 bp minimal target sequence named *FRT*, Flippase Recognition Target, with an *in vivo* efficiency of 100% 13 (Fig. 1). The FLP/*FRT* system has been widely utilized in many higher eukaryotes such as plants, *Caenorhabditis,* mice, somatic cell lines and in embryonic stem cells 14,15,16,17,18. In *Saccharomyces*, the recombination efficiency of FLP allowed the generation of a large number of applications such as a marker recycling system for multiple gene disruption 19 and “sticking” a gene wherever in the yeast genome (STIK = specific targeted integration of kanamycin resistance DNA) without a resident selective marker 20. Moreover, the FLP/*FRT* system was used specifically to induce translocations via homologous recombination in mitosis in *Drosophila* 21 and in *P. berghei* 22. In *Saccharomyces*, it is possible to obtain a translocation exploiting two *FRT*s sites cloned within two different chromosomes and expressing FLP transiently (under an inducible promoter) in a [*cir*^0^] (= lacking the 2µ plasmid) diploid strain. It is well know that a single mutation in the core region of the *FRT* abolishes the self-recombination with the wild type *FRT* in [*cir*^+^] strains and that the identity between the core regions of two *FRT* sites is necessary, but not sufficient for recombination in [*cir*^0^] strains 23. A modification of the FLP/*FRT* system can be used in multiple rounds of gene deletions, leaving scars that cannot recombine again between them also in [*cir*^+^] strains 20,24. These are major advantages of the FLP/*FRT* system if compared with other enzyme-dependent technologies that allow the construction of many different couples of identical recombination sites recombining between them, but not among them 19.

Few years after the discovery of FLP, another site-directed recombination system was explored 9. The bacteriophage P1 *CRE* gene was firstly expressed in tobacco cells 25, catalyzing deletion or inversion events when the *lox* sites were present in direct or inverted orientation respectively. The Cre/*lox* site-specific recombination system from bacteriophage P1 belongs to the tyrosine integrase family, it promotes the circularization of the bacteriophage after bacterial infection, and maintains the phage genome as unit-copy plasmid by resolving dimeric plasmids during bacterial division. Surprisingly, the minimal nucleotides length required for recombination of the *lox* (locus of crossing-over), 34 bp, is exactly the same as the one of the *FRT *(Fig. 1). Cre and FLP share some other features, but their comparison leads also to outline substantial differences between them. Cre/*lox* has been extensively used to direct DNA rearrangements in heterologous organisms also thanks to its natural thermo-stability at 37°C. The optimal temperature of action of the wild type recombinase FLP is of course lower (30°C), since it naturally functions in *Saccharomyces*. The development of a thermo-stable mutant of FLP, with four amino acids substitutions 26, overcame this drawback of the yeast flippase, extending its applications to mammalian cells. Cre has been reported to be toxic to cells 27 whereas FLP has never been reported to be toxic. Cre has a higher affinity for its target than FLP for *FRT*, both recombinases binding the two halves of the target cooperatively, while FLP has the highest excision efficiency and the highest recombinase-mediated cassette exchange rate 28. In yeast, the Cre/*loxP* has been extensively applied for industrial gene targeting 29, sequential gene deletion 30 and to generate gross chromosomal rearrangements. For instance, the system was used to reinforce the idea that translocation does not exactly drive speciation, but contributes to reproductive isolation between species of *Saccharomyces* *sensu stricto* yeasts 31. Afterward, the same authors concluded that, based on experiments of competition between species, reciprocal translocations obtained through the Cre/*lox* system, could have positive fitness and their position could be fixed by natural selection 32. It is interesting to note that cryptic *lox *sites (pre-existing pseudo-recognition sites) exist naturally in the *Saccharomyces* genome and that they can recombine among them even if with low frequency (( 1 x 10^-7^) 33. The intra-recombination of similar, but not identical *loxP *sites is an aspect to consider when choosing this targeting system. In fact, promiscuity of different *lox* sites can affect genome stability and intensive studies to generate *lox* mutants have been made so far trying to minimalize the problem 34,35.

Other site-specific recombination systems have been exploited in the past years to produce translocants: the Gin-gix system from bacteriophage Mu 10, the R-*RS *system from *Zygosaccharomyces rouxii* 11, the CinH-RS2 from *Acetinetobacter* 36, the ParA system from a plasmid operon parCBA 37 and the *Streptomyces* phage phiC31 system 38. One of the first discovered, the Gin recombinase, selectively mediates DNA recombination, but with the assistance of three additional factors: DNA negative supercoiling, an enhancer sequence and its binding protein Fis 39. Therefore, due to the increased technicality, its applications are limited. The most useful of these systems, if working with yeast cells, is the R-*RS*, because it operates efficiently in *Saccharomyces* [*cir*^+^] strains and independently from the host recombination system. pRS1 is a plasmid similar to 2µ that can replicate in *S.* *cerevisiae* as well as in its native host *Z. rouxii*. Reciprocal recombination between two non-homologous chromosomes were easily obtained with this method 11, although the presence of multiple copies of the RS alone (without the enzyme) might trigger further recombination events whose frequencies are of the same level as that caused by the Ty elements 11. RS sites must have a 7 bp core flanked by 12 bp inverted repeats; the asymmetric core sequence determines the site’s orientation and thus the types of recombination product (see Fig. 1). The R-recombinase has been shown to function in plants 40 where it catalyzes recombination between RSs that are either present extra-chromosomally in tobacco cells or stably resident in the tobacco chromosome with a 20% of estimated efficiency 40.

Among the other systems, the phiC31 from *Streptomyces*, tested in the fission yeast 41, but never in *Saccharomyces,* is currently under evaluation for translocation studies in higher eukaryotes; unfortunately, the unspecific translocation rate, due to integration within cryptic sites, may reach 15%, independently from the cell line used 42.

In conclusion, recombinase-dependent chromosomal rearrangements are widely utilized but need implementation to avoid spurious integrations and consequent secondary rearrangements among the target sequences in sequential rounds of transformations. Moreover, their utilization requires a time-consuming previous engineering of the host strain and, in few cases, the co-presence of additional factors. Unlike endogenous HR, enzyme-mediated recombination efficiency drops with increasing distance between recombination sites. Sometimes the expression level of the recombinases is difficult to be modulated and it reaches toxic levels. Natural hotspots widespread in the yeast genome may heavily affect the outcome of the targeted rearrangement, interfering with the specific recombination event. The strict sequence requirements imposed by site-specific recombinases have limited their applications to organisms that contain artificially-introduced recombination sites or natural pseudo-recognition sites. To address this limitation, laborious works of directed evolution have been used to alter the sequence specificity towards naturally occurring DNA sequences 43. Among many efforts, a combination of computational and experimental strategies has been considered to utilize native genomic sequences as FLP recombination target sites 44.

For all these drawbacks, targeted systems based merely on the endogenous homologous recombination machinery of *Saccharomyces* have contemporarily been studied and improved. These enzyme-independent site-specific methodologies will be described in details in the next paragraphs.

## ENZYME-INDEPENDENT SYSTEMS TO INDUCE REARRANGEMENTS IN THE YEAST GENOME: TAKING ADVANTAGE OF THE ENDOGENOUS MOLECULAR FACTORS

The yeast genome is relatively easy to manipulate thanks to its proficient homologous recombination system (HRS). The frequency of a homologous DNA targeting in *Saccharomyces* is considerably higher than an illegitimate integration and varies between 10^-4^ and 10^-6^ 45, depending on the length of the homology chosen, on the yeast strain and on the genomic locus selected as target. In effect, when working with budding yeast, we have always to keep in mind that a stretch of DNA homology of 40 nucleotides is enough to obtain a Rad52-dependent correct integration within the yeast genome with a minimal frequency, but that the accuracy of this process is strongly affected by at least two factors: the ploidy of the strain and the intrinsic recombinogenicity of the targeted genomic site. Indeed, ploidy dictates different repair pathway choice in diploid and haploid yeasts 46; moreover, the extension of at least two strain-related recombination hotspots (the rDNA region within chromosome XII and the sub-telomic sequences of each chromosome) deeply affects the outcome of the integration events at those sites. Furthermore, other widespread hot and cold spots of recombination are randomly distributed in the yeast genome and can additionally alter the rearrangements frequency 47,48. Nevertheless, *Saccharomyces* senses exogenous free ends as endogenous chromosomal fragment ends that result after a DSB, attempting a repair by homologous recombination (HR). The observation that free ends are highly recombinogenic in *Saccharomyces *allowed easy DNA-mediated manipulation of the yeast genome and its re-shaping.

Almost twenty years ago, few authors created an interesting methodology to restructure the yeast chromosomes 12. The introduction of a CFV into *S. cerevisiae* resulted in target homologous recombination events with consequent alteration in the strain’s karyotype. The CFVs contain *URA3 *as selectable marker, *SUP11* for visual monitoring of the chromosomal fragment stability based on the red/white Roman effect, sequences from the Y’ sub-telomeric repeat to generate a new yeast telomere (like in Yeast Artificial Vectors) and they carry a unique DNA segment of the yeast genome (= size of fragmentation: 1 - 5 Kb). The CVF is linearized, transformed into yeast and stable colonies are selected on -URA. The *in vivo* result is a chromosome fragment with a centromere, telomeres, and genomic sequences from the site of fragmentation chosen as target to the telomere. It was demonstrated that the chromosome fragment formation results from a replicative repair event, named “break-copy duplication” 12. The final outcome is a segmental aneuploidy due to a chromosome arm duplication initiated by each of the two targeting segments of the CFV. This simple experiment is one of the first examples on how it is possible to modify the karyotype of yeast exploiting endogenous molecular pathways such as the HRS and the replication complex.

In many recent works, the original DSB to generate free ends and provoke the repair demand is artificially produced by the cleavage of well-known homing endonucleases. The most used are the yeast-made I-SceI 49 and HO 50, which naturally have large cognate recognition sequences (18 - 24 nt) with a consequent extremely low probability of natural occurrence of their cleavage sites in the genomes (approximately 1.4 x 10^-11^). These endonuclease-based systems have been largely utilized to study homologous recombination 51, and micro-homology mediated repair in yeast cells 52.

Chromosomal translocations have been triggered via HRS thanks to these endonucleases functioning in yeast and in higher eukaryotes 53,54,55, but all these studies are based on the assumption that, after the HO/I-SceI cut, the free ends undergo an extensive process of 5’-(3’ DNA degradation until flanking regions of homology are exposed. Endonuclease-stimulated translocations are always formed through the non-conservative HR mechanism of single strand annealing 51, but it has never been completely elucidated how much other pathways, such as non-homologous end joining, can effectively compete for the break re-joining and how much a long (≥ 9 nt) stretch of non-homologous nucleotides can elicit other recombination pathways rather than the HRS.

A further bottleneck of these methodologies is that, as in the Cre/*loxp* and FLP/*FRT* systems, a previous engineering of the strain is necessary to introduce the endonuclease recognition sites and to induce their transcription. The regulatory network of inducible promoters in yeast (the most used is the galactose promoter) is extremely complex and differs significantly among the strains due to evolution and adaptive genome rewiring 56. In particular, few elements of the *GAL* system, such as the repressor Gal80 and the binding sites of the activator Gal4, often show spontaneous mutations 56. As consequence, gene expression induction level due to galactose addition retains sensitive differences among yeast strains. Other inducible promoter systems are activated by addition of common inducers, such as maltose or copper 57, and are largely used in *S. cerevisiae*. However, they are usually less inducible than galactose promoters, few of them are dependent on toxic inducers (i.e. copper) and give a background expression that is higher under non-repressing and non-inducing conditions (especially maltose). In industrial fermentation processes, the *ADH2-*related system is frequently used because its expression is turned on in stationary phase, when biomass is high, and does not require any specific inducer 57,58. Nevertheless, *ADH2* is several hundred-fold repressed in presence of glucose, and a de-repression mechanism with a trans-acting positive transcriptional effector is needed.

For all these reasons, in the last years, more naïve and portable systems that would work on any wild type yeast strain were deeply investigated, trying to avoid artifacts and addition of exogenous effectors. As a result, BIT technology was invented and will be discussed in the next paragraphs.

## HELPING A BIT TO GENERATE *ad hoc* CHROMOSOMAL REARRANGEMENTS

### From KARMA to BIT is a small step

More than ten years ago, we were studying the possibility to set up a simple system to detect mitotic recombination hotspots in the yeast genome. The idea was based on the observation that a sort of consensus came out aligning several known hotspot sequences (the majority of them meiosis-related). Using degenerated sequences named KARMA (from a short degeneration motif present within the consensus DNA sequence) we built by PCR a linear DNA cassette carrying a selectable marker and used it to transform yeast diploid cells. Unexpectedly, the KARMA cassette did not integrate in specific loci of the genome revealing hotspots, but led to aneuploidies. In fact, yeast cells preferred to consider the two free ends of the linear cassette as independent sequences, suitable for independent integration events. The result was chromosomal translocations with consequent ploidy variations.

**Figure 2:**
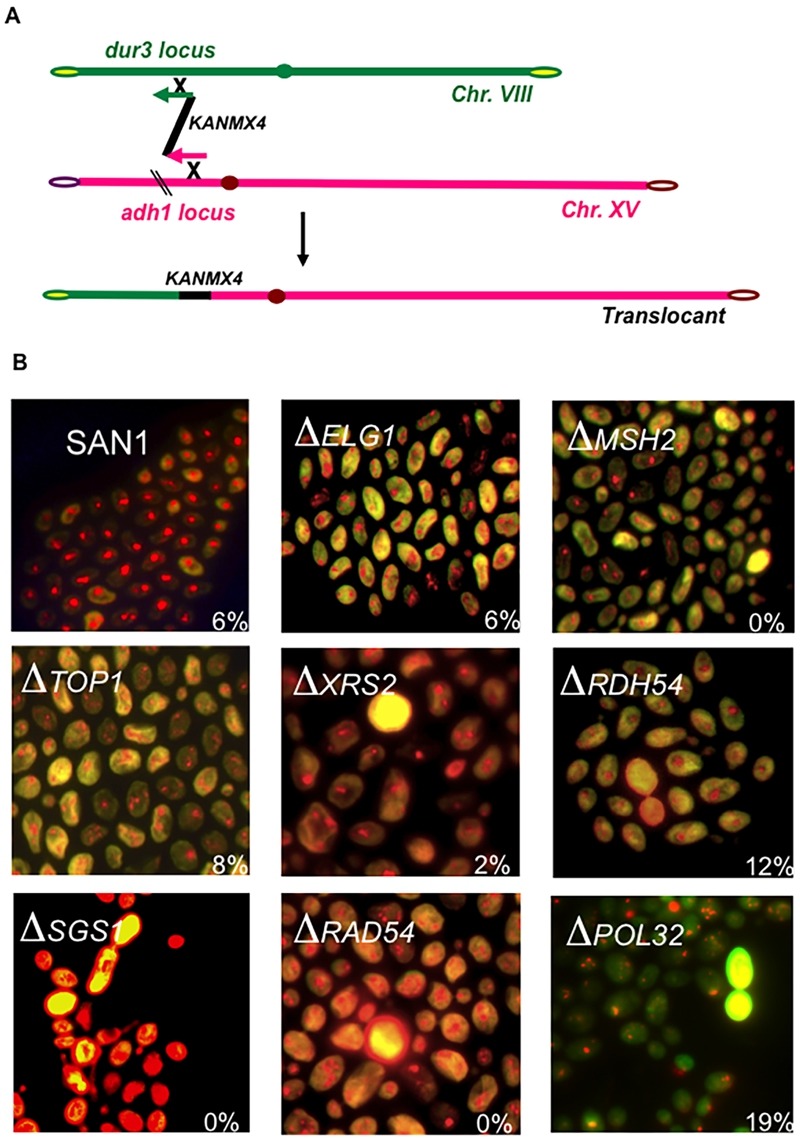
FIGURE 2: **(A)** Schematic representation of the BIT translocation between the *adh1 *locus (Chromosome XV) and the *dur3 *locus (Chromosome VIII); arrows at the end of the homology indicate the directionality of the translocation. **(B)** Fluorescent microphotography of the wild type yeast diploid SAN1 and several deletants of genes involved in the BIT regulatory pathway. The cells were stained with the Live/Dead Viability kit (Molecular Probes) using 6 μM as final dye concentration and fluorescein and rhodamin filters. Dead cells appear as yellow. The percentage of the translocants out of the total number of transformants obtained with the translocation represented in (A) is reported for each strain.

We decided to verify the hypothesis that the cell sensed two different homologies toward two different chromosomes as two independent events. The new DNA cassette, carrying a homology of 40 nt for chromosome VIII (locus* dur3*), another for chromosome XV (locus* adh1*) and kanamycin in between generated a non-reciprocal chromosomal translocation 59 (Fig. 2A). We verified soon that the system was easily reproducible, that it could be applied to every different genomic locus and that its efficiency was strongly affected by the length of the targeted homology. Using from 40 to 70 nucleotides as length of the recombinogenic ends and transforming approximately 2.2 - 3.4 x 10^8^ cells with 10 µg of linear DNA, the average frequency of the translocation, among all the transformants recovered, varied from 2 to 10%. The fluctuation in the frequency depends on the strain used and on the intrinsic recombinogenicity of the targeted genomic locus. Stable translocation-derived recombinants are generated among an ensemble of genomic rearrangements, comprising: i) intrachromosomal deletion, ii) ectopic integration (with a preference for the repeated rDNA region of chromosome XII), iii) aspecific translocations promoted by DNA micro-homology and, iv) recombination with the endogenous 2μ plasmid. In more than 30% of all transformants, only one of the two free ends with 40 nt of homology is correctly integrated via HRS at its homologous genomic site while the remaining majority show ectopic integrations. The results were also confirmed in trisomic strains suggesting that the phenomenon is not restricted to diploid cells 56. The system, named BIT, opened a Pandora’s box for successive discoveries.

### BIT elicits a local and a general effect on gene expression

We tested several genomic loci and different yeast strains, generating a collection of BIT translocants. Some translocants exhibited an abnormal cell phenotype with elongated buds as well as multibudded and unevenly nucleated cells, germination tube formation, pseudohyphal growth and nuclei fragmentation 60,61. The frequency of these morphological defects varied with the different translocation event, but usually represented the 2 - 20% of the total cell population after 40 h of growth on rich medium 61. After this time, the number of the abnormal cells, as well as of actively dividing cells, decreased in all strains, but the altered phenotype due to the G2/M arrest and to the low level of cyclin B1 persisted until the possible completion of the damage repair process 60. Other translocants revealed, through several Southern hybridizations 60,61, further genetic rearrangements of the acentric chromosome fragments generated in the translocation event, whereas expression of the genes located at the translocation breakpoints was increased up to five times, coinciding, in general, with an increased level of the RNA polymerase II binding to their promoters and to the pattern of histone acetylation 60,62. This so-called “local effect” on gene expression extended for at least 160 Kb from the breakpoint toward the telomeres. In addition to these local, *cis* effects, we also observed *trans* effects attributable to the deregulation of genes not physically involved in the chromosome translocation 60.

In some translocants, decreased cyclin B1 protein (but not its mRNA transcript) level could explain the appearance of cells with elongated buds, possibly arrested at the G2/M transition phase 60. The arrest was also confirmed by FACS analysis that indicated arrested cells in G1 and G2/M. Moreover, the expression of the actin 1 protein of the translocants and its deposition pattern was usually modified in agreement with abnormal cell morphology 60,61. These results indicate that a single translocations event can trigger a cascade of events, eventually ensuing in genomic instability and affecting general physiology of the cell 61,62.

Furthermore, it suggests that the translocant cells have adapted to the checkpoint response to the initial DNA damage generated by BIT chromosome translocation. In fact, besides the DSBs, necessary for the homologous DNA integration, BIT produces extensive genomic damages that, thanks to the signal transduction pathway of the damage checkpoint system, triggers an impairment-induced G2/M arrest until completion of the repair. However, after the translocation event, the cells do not remain permanently arrested, but resume progression through the cell cycle 60. The escape from G2/M arrest occurs despite the broken chromosomes and the translocants will eventually override this checkpoint adapting to the new chromosomally aberrant condition 63. Two typical physiological and biochemical landmarks, a decreasing activity of Rad53 and the absence of its phosphorylation 64, confirm adaptation in BIT translocants 60.

The concept that a heterogeneous phenotypic population may arise from a single translocation event was analyzed in details 61, characterizing ten morphologically and phenotypically different translocants resulting from the same BIT event in *S. cerevisiae.* At least eight different pathways were exploited to heal the broken chromosome and allow survival. Endo-reduplication of both chromosomes was possible, leading to strains trisomic for chromosome XVI, tetraploid for chromosome IX, or more complex rearrangements whose outcome was a partial duplication of one of the two chromosomes through a template switching - like mechanism 61.

The ploidy condition and its related phenotype are unique for each translocant as unique is its transcription pattern 61. It seems that the loss of a predefined chromosomal homeostasis, while affecting the strain identity, does not impair its vitality; it was in fact verified that few translocants show an improved fitness and gained resistance to drugs and metabolites 62.

We speculated whether the same deregulation could happen when performing BIT between homologous chromosomes in diploid cells. The next paragraph will focus on this topic.

### BIT between heterologous and homologous chromosomes: similarities and differences in patching chromosomes 

The main advantage of BIT is that specific translocations can be generated *ad hoc* without genetically engineering the strains, simulating the situation of *in vivo* linear DNA - induced GCRs. We wondered if this system could be applied to bridge together two homologs in a diploid yeast cell. Bridging together two homologous chromosomes VIII of *S. cerevisiae,* it was found i) that the specificity and the efficiency of gene targeting is decreased with the increasing of the distance between the two homologies within the genome, ii) that the occurrence of targeted BIT between homologous chromosomes is lower than between heterologous, and iii) that nearly - reciprocal translocants are formed with the loss of genetic material that could provoke the loss of heterozygosity (LOH) 24.

The phenotypical and transcriptional aberrations of the translocant between homologues are negligible if compared with those of non-reciprocal translocants between different chromosomes. Moreover, the quantitative analysis of the expression of several genes, located within 80 Kb of chromosome VIII around the translocation point, did not detect any significant increase in expression with the exception of multidrug resistance genes. The nearly - reciprocal translocation is probably due to a migration of the holliday junction followed by a recession of the homology with consequent LOH (for a model see Fig. 4 from 24). These results support our idea that the formation of a novel chromosome after BIT between heterologous chromosomes and not the possible partial loss of a chromosomal arm in diploid cells is responsible for the strong genetic deregulation and the consequent phenotypic aberrations we observed in the translocants. In fact, few translocants, retaining and rearranging the acentric fragments generated by a BIT event, show some of the worst phenotypes usually associated with heavy karyocinetic defects 59,60. Moreover, the final result of a translocation between homologous chromosomes is a DNA deletion with LOH and not a new chromosome formation since the two starting homologs are basically identical. If we hypothesize that self-determinant elements exist in each chromosome, it is not surprising that a near-reciprocal translocant strain between two homologs maintains the same gene expression levels it had before the translocation event, assuming that the identity element has been retained at least on one of the two homologs.

The directionality of migration of the holliday junction after BIT between homologs can be strongly affected by the action of Rdh54 65. Rdh54 (Rad54B in mammals) promotes branch migration and unwinds three-strand DNA structures and has a role in holliday junction translocation during BIT events. Its absence or impairment dramatically changes the outcome resulting from a translocation between homologs controlling the amount of heterozygosity between the two ensuing homologs 65. The low efficiency (less than 1%) of BIT between homologous chromosomes confirms the idea that an active system exists to prevent the instability of the genome, avoiding that endogenous or exogenous linear DNA such as transposons and mitochondrial DNA fragments could engender mitotic rearrangements. Searching for the molecular players responsible for this strict enzymatic control, we discovered that chromosomal translocations triggered by BIT are strongly Rad4-dependent and Pol32-independent. Pol32 is responsible for many events of the previously cited break-copy duplications to generate CFV. This pathway was then re-named break induced replication (BIR) although it was never demonstrated to be exactly the same phenomenon 66; more recently Deem and co-workers found that some Pol32-independent BIR could be also observed in yeast 67. The bridge completion during BIT is an example of these events. After the construction in a yeast diploid strain of double deletants of genes already known to be involved in chromosomal translocations (Fig. 2B), we discovered that many factors are involved in BIT regulation 67. Among them, Rdh54 is essential in determining the LOH outcome in translocations between homologs and important in the stability of the translocated chromosome in translocations between heterologous chromosomes 65.

**Figure 3:**
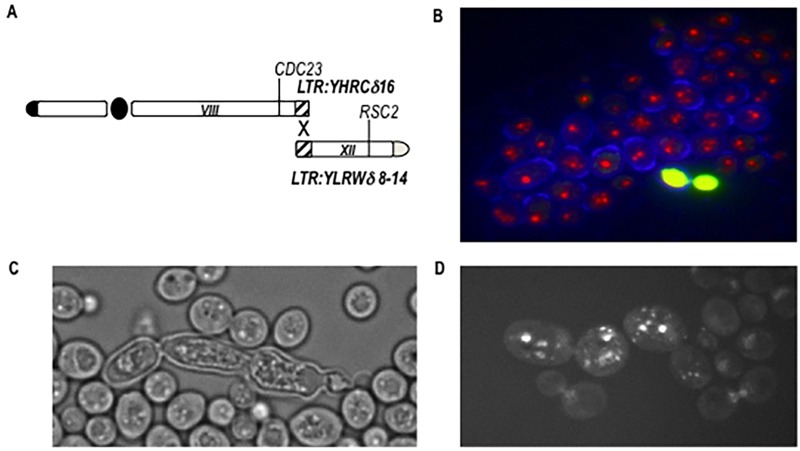
FIGURE 3: Analysis of a BIT-translocant over-expressing *RAD54*. **(A)** Scheme of a further rearrangement event following a primary BIT translocation (represented in Fig. 2A). Two almost identical LTRs, one on the right arm of chromosome VIII and another on the right arm of chromosome XII, recombine leading to a new translocation between chromosome VIII and XII (scheme adapted from 65). **(B)** In BIT translocants over-expressing *RAD54*, many dead cells are present. Live/Dead staining (Molecular Probe), here combined with Calcofluor, shows the presence of dead cells (in yellow). **(C)** Abnormal phenotype of dead BIT translocants over-expressing *RAD54.* (D) DAPI staining indicates severe nuclei fragmentation when *RAD54* is over-expressed in BIT translocants.

One of the most interesting observations is on the role of Rad54 in chromosomal translocations. Its absence, as predictable, eliminates the possibility of translocation through the HRS. On the contrary, the over-expression of *RAD54* triggers secondary rearrangements between long-terminal repeats (LTRs) located far from the breaks, producing an array of further multiple translocations. A schematic summary of the multiple rearrangements deduced from twelve different hybridizations is represented in Fig. 3A. In the case of a BIT translocation between chromosome VIII and XV, we verified that two almost identical LTRs (one on chromosome VIII and the other on chromosome XII) were recombining after the initial BIT event in translocants over-expressing* RAD54 *and that these further rearrangements might correlate with an abnormal phenotype and poor cell viability (Fig. 3B, C, D). Rearrangements between LTRs as well as abnormal phenotypes were present in all the translocants over-expressing *RAD54* and resulted stable once formed 67. This discovery could yield insights into the key role of the HR player *RAD54* in break-distal rearrangements between repeats, which are landmark traits of tumor cells. Many chromatin remodelers and in particular the SWI2/SNF2 family of ATPase-dependent DNA translocases, have been correlated to ploidy maintenance 68 and, among these, *RAD54* is found over-expressed up to 5-fold in prostatic cancer cells characterized by recurrent non-reciprocal translocations 69. We can conclude that the stoichiometric balance of Rad54 is essential for an efficient post-synaptic phase of the HR between exogenous linear DNA and the yeast genome.

## CHROMOSOMAL TRANSLOCATIONS ELICIT ACTIN NETWORK DEREGULATION AND MIGHT RESULT IN MORPHOLOGICAL SWITCH

**Figure 4:**
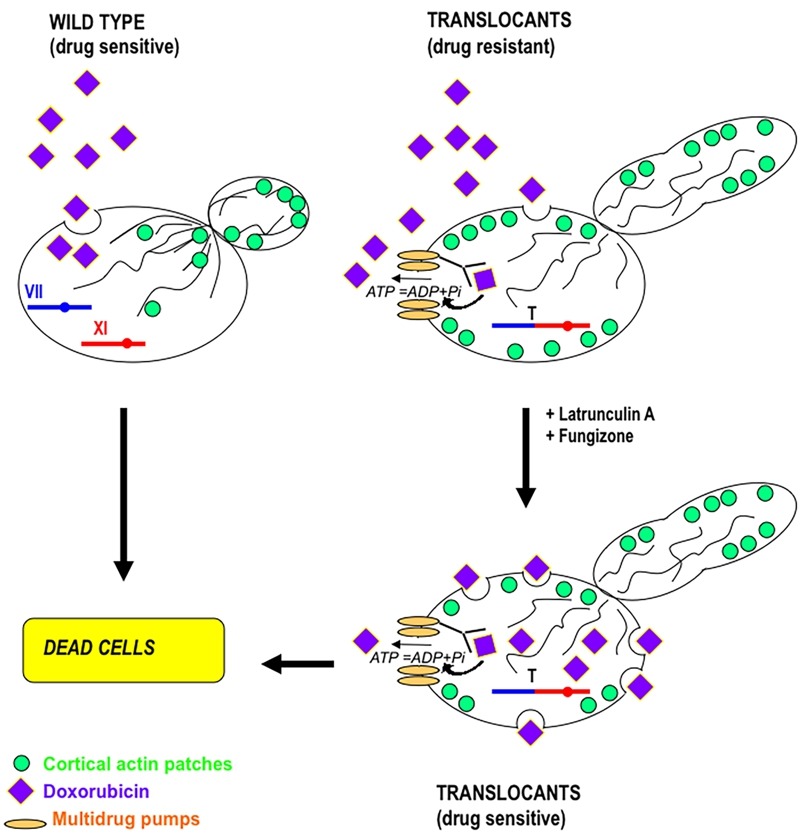
FIGURE 4: A schematic representation of the molecular mechanism for drug-resistance in diploid yeast translocant strains over-expressing *PRK1* and *PDR* genes. The effect of doxorubicin on a wild type strain and on a translocant strain is compared. Doxorubicin can easy enter into a wild type yeast strain, killing it. On the contrary, due to the disaggregation of actin cortical patches and to over-expression of multidrug pumps, the translocants become resistant to doxorubicin. The addition of sub-lethal doses of fungizone and latrunculin A allows bypassing the drug resistance and makes yeast translocants even more sensitive to doxorubicin 62. Here, a hypothetical translocation between chromosome VII and chromosome XI is represented. For illustration purposes, only one chromosome (the one involved in the translocation) of each pair is drawn.

In several yeasts morphological changes that correspond to acquired drug resistance, can be associated to chromosomal translocations 70,71. This hetero-resistance can become a serious clinical problem in the case of pathogenic yeasts such as *Candida spp*. and *Cryptococcus neoformans* 72,73. If the development of azole resistance in *C. albicans* associated with point mutations in the *ERG11* gene has been well documented 74, the translocation-dependent azole resistance has never been clearly elucidated. It is known that deletion of some of the HRS genes leads to an increased susceptibility to antifungal drugs 72 and that the non homologous end joining gene *LIG4* does not contributes to adaptive chromosomal translocations 71. The appearance of isochromosomes in *C. albicans *75 and of translocation-dependent small chromosomes in *C. glabrata* is responsible for an increased fitness and drug resistance 70. Several HRS mutants both in *Saccharomyces* and in *Candida* show pseudo-hypha formation 71,72, while wild type strains respond often to environmental and nutritional stress with a yeast-to-filaments morphogenetic switch. The regulatory network that controls budding yeast filamentation remains under investigation even after it was demonstrated that nuclear export of the osmoresponsive MAPK Hog1p may enhance pseudohyphal growth 76. Furthermore, it is known that BIT-induced chromosomal translocants show karyocinetic defects, hyphal development and increased resistance to anticancer chemicals like Doxorubicin and Latrunculin A due to an endocytic actin network deregulation 62. The actin deregulation is prompted by over-expression of the *PRK1 *serine/threonine protein kinase gene. Other genes belonging to the ABC transporters group, such as *PDR1*, *PDR3 *and *VMR1*, are sometimes over-transcribed in budding yeast carrying translocations between heterologous 62 and homologous chromosomes 24, contributing to pleiotropic drug resistance (see Figure 4 for a summary of the drug resistance molecular process). It is important to note that aneuploidies due to translocations generate drug-resistant variants by chromosome reassortments and karyotype instability not only in yeast, but also in cancer cells 77.

Therefore, it seems that in both *Saccharomyces* and *Candida* the HRS-dependent chromosomal translocations might result in morphological alterations and adaptive mutagenesis. On the contrary, the non homologous end joining pathway does not appear to be involved in adaptations mediated by ploidy variation 71. Moreover, translocations lead to appearance of cells with varied levels of resistance to many compounds and when some selection is applied to these cells, the most resistant in the population will obviously survive. In this case, chromosomal translocations could act as genetic basis of variability for complex molecular adaptation to changed environmental conditions, leading, for example, to increased drug resistance of human pathogenic yeasts 70 or to the origin of new species 32.

## POST-TRANSLOCATIONAL ADAPTATION (PTA) TO THE CHECKPOINT RESPONSE

Aneuploid strains are generally characterized by strong genomic instability that may result from imbalances in particular genes and/or from proteotoxic stress 78. This genomic instability, while providing great advantages to microorganisms’ evolution is also the origin of tumors in mammals. After the induction of chromosomal translocation, the yeast cells undergo an adaptation following a G1 or a G2/M arrest 60. Many of these translocants are highly unstable and are prone to loose the extra-chromosome or to generate further secondary rearrangements 65. The recombination between repetitive sequences is generally increased, and the possibility of further aneuploidies arises 65. The genome instability and DNA repair defects are usually associated to an excess protein (such as cyclin B1, 60) and to stoichiometric imbalances 79. Even if chromosomal translocations confer severe disadvantages to the cells such as an altered metabolism and homeostasis and represent a real danger during damage repair, they could be a benefit in terms of competitive growth and development. Both reciprocal 32 and non-reciprocal translocations 62 can have positive-fitness effects resulting in an increased growth rate in *Saccharomyces*. For example, from a biotechnological point of view, yeast strains adapted to cold temperatures during wine fermentations are needed by the industry because the wine aroma is improved at low temperatures. Recent results indicate that these adapted yeasts show proteomic changes due to a greater activation of some metabolic routes such as glutathione biosynthesis and sulfur assimilation 79,80. Since the majority of wine yeast strains are aneuploid, we believe that their metabolic deregulation might be due to a post-translocational adaptation to the fermentation conditions.

This hypothesis is supported by our recent unpublished observations on BIT translocants that may acquire some complex phenotypic traits because of genes over-transcription following genetic selection (Tosato, personal communication). Adaptation might be also due to antagonistic pleiotropy, the ability of a specific gene to perform differently in different phenotypic conditions. Antagonistic pleiotropy was initially proposed as an explanation for aging and could be seen as adaptive evolution when the same genes are responsible for vitality and reproduction in cellular earlier life but a detriment at cellular later life 81. Nevertheless, it has been recently verified that antagonistic pleiotropy is practically absent in natural yeast isolates probably through accumulation of compensatory mutations 82. Thus again, it enforces the idea that GCRs and not antagonistic pleiotropy have a fundamental role in balancing fitness over the course of evolution.

It was already known that Ty-dependent recombination might have a beneficial effect in the long term, restructuring the yeast genome architecture 83 and leading to a faster temporary adaptation to the environment 84. The number of LTRs fluctuates approximately around mean of 300, with a conserved sequence of ≈ 332 bp. Like the repetitive human elements, Ty1s appear to mediate many types of rearrangements, including inversions, deletions, and both reciprocal and non-reciprocal translocations. Ty recombination is rare and, most importantly, restricted to the haploid state. Here we propose that not only transposon recombination in haploids, but also HR-driven recombination in diploids, due to any exogenous or endogenous homologous linear DNA, can trigger translocations, multiple secondary rearrangements, then genome instability and consequent adaptation, providing a quantum leap in genomic evolution.

## CONCLUSIONS

As conclusive remarks, chromosomal translocations can become a useful model to mimic chromosome reassortments catalyzed by aneuploidy in cancer cells and to optimize biotechnological products through the selection of beneficial properties of new strains. These studies may result in important industrial outcomes providing novel chemotherapy adjuvants and unique foodstuff manufacturing processes on the market.

New strains are the final upshot of a genome remodeling due to a translocation. The strains coming from the same translocation event are all different because different is the answer to a destabilizing event in terms of transcriptional genetic alteration and pathway choice to heal the broken chromosome. The resulting strains are usually impaired and rarely improved in fitness, having in this last case the chance to survive, progress and develop. They have reached a novel stable advantageous condition (*astra*) through adaptation and difficulties (*aspera*). In one word, through some sort of trial and error, they have evolved.

## References

[1] Zimmer A, Durand C, Loira N, Durrens P, Sherman DJ, Marullo P (2014). QTL dissection of Lag phase in wine fermentation reveals a new translocation responsible for Saccharomyces cerevisiae adaptation to sulfite.. PLoS One.

[2] Luo Z, van Vuuren HJ (2009). Functional analyses of PAU genes in Saccharomyces cerevisiae.. Microbiology.

[3] Codon AC, Benítez T, Korhola M (1998). Chromosomal polymorphism and adaptation to specific industrial environments of Saccharomyces strains.. Appl Microbiol Biotechnol.

[4] Brion C, Ambroset C, Sanchez I, Legras JL, Blondin B (2013). Differential adaptation to multi-stressed conditions of wine fermentation revealed by variations in yeast regulatory networks.. BMC Genomics.

[5] Iwaguchi SI, Kanbe T, Tohne T, Magee PT, Suzuki T (2000). High-frequency occurrence of chromosome translocation in a mutant strain of Candida albicans by a suppressor mutation of ploidy shift.. Yeast.

[6] Dunham MJ, Badrane H, Ferea T, Adams J, Brown PO, Rosenzweig F, Botstein D (2002). Characteristic genome rearrangements in experimental evolution of Saccharomyces cerevisiae.. Proc Natl Acad Sci U S A.

[7] Rachidi N, Barre P, Blondin B (1999). Multiple Ty- mediated chromosomal translocations lead to karyotype changes in a wine strain of S. cerevisiae. Molecular and General Genetics.

[8] Andrews BJ, Proteau GA, Beatty LG, Sadowski PD (1985). The FLP recombinase of the 2 micron circle DNA of yeast: interaction with its target sequences.. Cell.

[9] Dale EC, Ow DW (1990). Intra- and intermolecular site-specific recombination in plant cells mediated by bacteriophage P1 recombinase.. Gene.

[10] Kahmann R, Rudt F, Koch C, Mertens G (1985). G inversion in bacteriophage Mu DNA is stimulated by a site within the invertase gene and a host factor.. Cell.

[11] Matsuzaki H, Nakajima R, Nishiyama J, Araki H, Oshima Y (1990). Chromosome engineering in Saccharomyces cerevisiae by using a site-specific recombination system of a yeast plasmid.. Proc Natl Acad Sci USA.

[12] Morrow DM, Connelly C, Hieter P (1996). “Break copy” duplication: a model for chromosome fragment formation in Saccharomyces cerevisiae.. Genetics.

[13] Ringrose L, Lounnas V, Ehrlich L, Buchholz F, Wade R, Stewart AF (1998). Comparative kinetic analysis of FLP and cre recombinases: mathematical models for DNA binding and recombination.. J Mol Biol.

[14] Akbudak MA, Srivastava V (2011). Improved FLP recombinase, FLPe, efficiently removes marker gene from transgene locus developed by Cre-lox mediated site-specific gene integration in rice.. Mol Biotechnol.

[15] Hubbard EJ (2014). FLP/FRT and Cre/lox recombination technology in C. elegans.. Methods.

[16] Bailey JM, Creamer BA, Hollingsworth MA (2009). What a fish can learn from a mouse: principles and strategies for modeling human cancer in mice.. Zebrafish.

[17] Schönhuber N, Seidler B, Schuck K, Veltkamp C, Schachtler C, Zukowska M, Eser S, Feyerabend TB, Paul MC, Eser P, Klein S, Lowy AM, Banerjee R, Yang F, Lee CL, Moding EJ, Kirsch DG, Scheideler A, Alessi DR, Varela I, Bradley A, Kind A, Schnieke AE, Rodewald HR, Rad R, Schmid RM, Schneider G, Saur D (2014). A next-generation dual-recombinase system for time- and host-specific targeting of pancreatic cancer.. Nat Med.

[18] Kuehle J, Turan S, Cantz T, Hoffmann D, Suerth JD, Maetzig T, Zychlinski D, Klein C, Steinemann D, Baum C, Bode J, Schambach A (2014). Modified lentiviral LTRs allow Flp recombinase-mediated cassette exchange and in vivo tracing of "factor-free" induced pluripotent stem cells.. Molecular Therapy.

[19] Storici F, Coglievina M, Bruschi CV (1999). A 2-micron DNA-based marker recycling system for multiple gene disruption in the yeast Saccharomyces cerevisiae.. Yeast.

[20] Waghmare SK, Caputo V, Radovic S, Bruschi CV (2003). Specific targeted integration of kanamycin resistance-associated non selectable DNA in the genome of the yeast Saccharomyces cerevisiae.. BioTechniques.

[21] Beumer KJ, Pimpinelli S, Golic KG (1998). Induced chromosomal exchange directs the segregation of recombinant chromatids in mitosis of Drosophila.. Genetics.

[22] Lacroix C, Giovannini D, Combe A, Bargieri DY, Späth S, Panchal D, Tawk L, Thiberge S, Carvalho TG, Barale JC, Bhanot P, Ménard R (2011). FLP/FRT- mediated conditional mutagenesis in pre-erythrocytic stages of Plasmodium berghei.. Nat Protoc.

[23] Storici F, Bruschi CV (1997). Molecular engineering with the FRT sequence of the yeast 2microm plasmid cir0 segregant enrichment by counterselection for 2 microm site-specific recombination.. Gene.

[24] Tosato V, 24 C, Bruschi CV (2009). DNA bridging of yeast chromosomes VIII leads to near-reciprocal translocation and loss of heterozygosity with minor cellular defects.. Chromosoma.

[25] Odell J, Caimi P, Sauer B, Russell S (1990). Site-directed recombination in the genome of transgenic tobacco.. Mol Gen Genet.

[26] Buchholz F, Angrand PO, Stewart AF (1998). Improved properties of FLP recombinase evolved by cycling mutagenesis.. Nat Biotechnol.

[27] Loonstra A, Vooijs M, Beverloo HB, Allak BA, van Drunen E, Kanaar R, Berns A, Jonkers J (2001). Growth inhibition and DNA damage induced by Cre recombinase in mammalian cells.. Proc Natl Acad Sci USA.

[28] Takata Y, Kondo S, Goda N, Kanegae Y, Saito I (2011). Comparison of efficiency between FLPe and Cre for recombinase-mediated cassette exchange in vitro and in adenovirus vector production.. Genes Cells.

[29] Qiu Z, Deng Z, Tan H, Zhou S, Cao L (2015). Engineering the robustness of Saccharomyces cerevisiae by introducing bifunctional glutathione synthase gene.. J Ind Microbiol Biotechnol.

[30] Hegemann JH, Heick SB, Pöhlmann J, Langen MM, Fleig U (2014). Targeted gene deletion in Saccharomyces cerevisiae and Schizosaccharomyces pombe.. Methods Mol Biol.

[31] Delneri D, Colson I, Grammenoudi S, Roberts IN, Louis EJ, Oliver SG (2003). Engineering evolution to study speciation in yeasts.. Nature.

[32] Colson I, Delneri D, Oliver SG (2004). Effects of reciprocal chromosomal translocations on the fitness of Saccharomyces cerevisiae.. EMBO Rep.

[33] Sauer B (1992). Identification of cryptic lox sites in the yeast genome by selection for Cre-mediated chromosome translocations that confer multiple drug resistance.. J Mol Biol.

[34] Missirlis PI, Smailus DE, Holt RA (2006). A high-throughput screen identifying sequence and promiscuity characteristics of the loxP spacer region in Cre-mediated recombination.. BMC Genomics.

[35] Langer SJ, Ghafoori AP, Byrd M, Leinwand L (2002). A genetic screen identifies novel non-compatible loxP sites.. Nucleic Acids Res.

[36] Moon HS, Abercrombie LL, Eda S, Blanvillain R, Thomson JG, Ow DW, Stewart CN Jr (2011). Transgene excision in pollen using a codon optimized serine resolvase CinH-RS2 site-specific recombination system.. Plant Mol Biol.

[37] Thomson JG Yau YY, Blanvillain R, Nunes WM, Chiniquy D, Thilmony R, Ow DW (2009). ParA resolvase catalyzes site-specific excision of DNA from the Arabidopsis genome.. Transgenic Res.

[38] Kittiwongwattana C, Lutz K, Clark M, Maliga P (2007). Plastid marker gene excision by the phiC31 phage site-specific recombinase.. Plant Mol Biol.

[39] Klippel A, Cloppenborg K, Kahmann R (1988). Isolation and characterization of unusual gin mutants.. EMBO J.

[40] Onouchi H, Yokoi K, Machida C, Matsuzaki H, Oshima Y, Matsuoka K, Nakamura K, Machida Y (1991). Operation of an efficient site-specific recombination system of Zygosaccharomyces rouxii in tobacco cells.. Nucleic Acids Research.

[41] Thomason LC, Calendar R, Ow DW (2001). Gene insertion and replacement in Schizosaccharomyces pombe mediated by the Streptomyces bacteriophage phiC31 site-specific recombination system.. Mol Genet Genomics.

[42] Ehrhardt A, Engler JA, Xu H, Cherry AM, Kay MA (2006). Molecular analysis of chromosomal rearrangements in mammalian cells after phiC31-mediated integration.. Hum Gene Ther.

[43] Buchholz F, Stewart AF (2001). Alteration of Cre recombinase site specificity by substrate-linked protein evolution.. Nat Biotechnol.

[44] Bolusani S, Ma CH, Paek A, Konieczka JH, Jayaram M, Voziyanov Y (2006). Evolution of variants of yeast site-specific recombinase Flp that utilize native genomic sequences as recombination target sites.. Nucleic Acids Res.

[45] Wach A, Brachat A, Pöhlmann R, Philippsen P (1994). New heterologous modules for classical or PCR-based gene disruptions in Saccharomyces cerevisiae.. Yeast.

[46] Li XC, Tye BK (2011). Ploidy dictates repair pathway choice under replication stress.. Genetics.

[47] Liu G, Liu J, Cui X, Cai L (2012). Sequence-dependent prediction of recombination hotspots in S. cerevisiae.. J Theor Biol.

[48] Jiang P, Wu H, Wei J, Sang F, Sun X, Lu Z (2007). RF-DYMHC: detecting the yeast meiotic recombination hotspots and coldspots by random forest model using gapped dinucleotide composition features.. Nucleic Acids Res.

[49] Colleaux L, d'Auriol L, Betermier M, Cottarel G, Jacquier A, Galibert F, Dujon B (1986). Universal code equivalent of a yeast mitochondrial intron reading frame is expressed into E. coli as a specific double strand endonuclease.. Cell.

[50] Nickoloff JA, Chen EY, Heffron F (1986). A 24-base-pair DNA sequence from the MAT locus stimulates intergenic recombination in yeast.. Proc Natl Acad Sci U S A.

[51] Liddell L, Manthey G, Pannunzio N, Bailis A (2011). Quantitation and analysis of the formation of HO-endonuclease stimulated chromosomal translocations by single-strand annealing in Saccharomyces cerevisiae.. J Vis Exp.

[52] Villarreal DD, Lee K, Deem A, Shim EY, Malkova A, Lee SE (2012). Microhomology directs diverse DNA break repair pathways and chromosomal translocations.. PLoS Genet.

[53] Haber JE, Leung WY (1996). Lack of chromosome territoriality in yeast: promiscuous rejoining of broken chromosome ends.. Proc Natl Acad Sci USA,.

[54] Egli D, Hafen E, Schaffner W (2004). An efficient method to generate chromosomal rearrangements by targeted DNA double-strand breaks in Drosophila melanogaster.. Genome Res.

[55] Gostissa M, Schwer B, Chang A, Dong J, Meyers RM, Marecki GT, Choi VW, Chiarle R, Zarrin AA, Alt FW (2014). IgH class switching exploits a general property of two DNA breaks to be joined in cis over long chromosomal distances.. Proc Natl Acad Sci USA.

[56] David L, Ben-Harosh Y, Stolovicki E, Moore LS, Nguyen M, Tamse R, Dean J, Mancera E, Steinmetz LM, Braun E (2013). Multiple genomic changes associated with reorganization of gene regulation and adaptation in yeast.. Mol Biol Evol.

[57] Shen MWY, Fang F, Sandmeyer S, Da Silva NA (2012). Development and characterization of a vector set with regulated promoters for systematic metabolic engineering in S. cerevisiae.. Yeast.

[58] Lee KM, Da Silva NA (2005). Evaluation of the S. cerevisiae ADH2 promoter for protein synthesis.. Yeast.

[59] Tosato V, Waghmare SK, Bruschi CV (2005). Non-reciprocal chromosomal bridge-induced translocation (BIT) by targeted DNA integration in yeast.. Chromosoma.

[60] Nikitin D, Tosato V, Zavec AB, Bruschi CV (2008). Cellular and molecular effects of non reciprocal chromosome translocations in Saccharomyces cerevisiae.. Proc Natl Acad Sci U S A.

[61] Rossi B, Noel P, Bruschi CV (2010). Different aneuploidies arise from the same bridge-induced chromosomal translocation event in Saccharomyces cerevisiae. Genetics.

[62] Nikitin DV, Bruschi CV, Sims J, Breitenbach M, Rinnerthaler M, Tosato V (2014). Chromosome translocation may lead to PRK1-dependent anticancer drug resistance in yeast via endocytiv actin network deregulation.. Eur J Cell Biol.

[63] Pedersen B, Nørgaard JM, Pedersen BB, Clausen N, Rasmussen IH, Thorling K (2000). Many unbalanced translocations show duplication of a translocation participant. Clinical and cytogenetic implications in myeloid hematologic malignancies.. Am J Hemat.

[64] Pellicioli A, Lee SE, Lucca C, Foiani M, Haber J (2001). Regulation of Saccharomyces Rad53 Checkpoint kinase during adaptation from DNA damage-induced G2/M arrest.. Mol Cell.

[65] Tosato V, Sidari S, Bruschi CV (2013). Bridge-induced chromosome translocation in yeast relies upon a Rad54/Rdh54-dependent, Pol32-independent pathway.. PLoS One.

[66] Lydeard JR, Jain S, Yamaguchi M, Haber JE (2007). Break-induced replication and telomerase-independent telomere maintenance require Pol32.. Nature.

[67] Deem A, Barker K, Vanhulle K, Downing B, Vayl A, Malkova A (2008). Defective break-induced replication leads to half-crossovers in Saccharomyces cerevisiae.. Genetics.

[68] Campsteijn C, Wijnands-Collin AM, Logie C (2007). Reverse genetic analysis of the yeast RSC chromatin remodeler reveals a role for RSC3 and SNF5 homolog 1 in ploidy maintenance.. PLoS Genet.

[69] Bristow RG, Ozcelik H, Jalali F, Chan N, Vesprini D (2007). Homologous recombination and prostate cancer: a model for novel DNA repair targets and therapies.. Radiother Oncol.

[70] Ahmad KM, Ishchuk OP, Hellborg L, Jørgensen G, Skvarc M, Stenderup J, Jørck-Ramberg D, Polakova S, Piškur J (2013). Small chromosomes among Danish Candida glabrata isolates originated through different mechanisms.. Antonie Van Leeuwenhoek.

[71] Andaluz E, Ciudad T, Larriba G (2002). An evaluation of the role of LIG4 in genomic instability and adaptive mutagenesis in Candida albicans.. FEMS Yeast Res.

[72] Legrand M, Chan CL, Jauert PA, Kirkpatrick DT (2007). Role of DNA mismatch repair and double-strand break repair in genome stability and antifungal drug resistance in Candida albicans.. Eukaryot Cell.

[73] Ngamskulrungroj P, Chang Y, Hansen B, Bugge C, Fischer E, Kwon-Chung KJ (2012). Characterization of the chromosome 4 genes that affect fluconazole-induced disomy formation in Cryptococcus neoformans.. PLoS One.

[74] White TC, Holleman S, Dy F, Mirels LF, Stevens DA (2002). Resistance mechanisms in clinical isolates of Candida albicans.. Antimicrob Agents Chemother.

[75] Selmecki A, Forche A, Berman J (2006). Aneuploidy and isochromosome formation in drug-resistant Candida albicans.. Science.

[76] Shively CA, Eckwahl MJ, Dobry CJ, Mellacheruvu D, Nesvizhskii A, Kumar A (2013). Genetic networks inducing invasive growth in Saccharomyces cerevisiae identified through systematic genome-wide overexpression.. Genetics.

[77] Duesberg P, Stindl R, Hehlmann R (2001). Origin of multidrug resistance in cells with and without multidrug resistance genes: chromosome reassortments catalyzed by aneuploidy.. Proc Natl Acad Sci USA.

[78] Sheltzer JM, Blank HM, Pfau SJ, Tange Y, George BM, Humpton TJ, Brito IL, Hiraoka Y, Niwa O, Amon A (2011). Aneuploidy drives genomic instability in yeast.. Science.

[79] García-Ríos E, López-Malo M, Guillamón JM (2014). Global phenotypic and genomic comparison of two Saccharomyces cerevisiae wine strains reveals a novel role of the sulfur assimilation pathway in adaptation at low temperature fermentations.. BMC Genomics.

[80] Paget CM, Schwartz JM, Delneri D (2014). Environmental systems biology of cold-tolerant phenotype in Saccharomyces species adapted to grow at different temperatures.. Mol Ecol.

[81] Williams GC (1957). Pleiotropy, natural selection, and the evolution of senescence.. Evolution.

[82] Yadav A, Radhakrishnan A, Bhanot G, Sinha H (2015). Differential Regulation of Antagonistic Pleiotropy in Synthetic and Natural Populations Suggests Its Role in Adaptation.. G3 (Bethesda).

[83] Chan JE, Kolodner RD (2011). A genetic and structural study of genome rearrangements mediated by high copy repeat Ty1 elements.. PLoS Genet.

[84] Carreto L, Eiriz MF, Gomes AC, Pereira PM, Schuller D, Santos MA (2008). Comparative genomics of wild type yeast strains unveils important genome diversity.. BMC Genomics.

